# JUMPm: A Tool for Large-Scale Identification of Metabolites in Untargeted Metabolomics

**DOI:** 10.3390/metabo10050190

**Published:** 2020-05-12

**Authors:** Xusheng Wang, Ji-Hoon Cho, Suresh Poudel, Yuxin Li, Drew R. Jones, Timothy I. Shaw, Haiyan Tan, Boer Xie, Junmin Peng

**Affiliations:** 1 Center for Proteomics and Metabolomics, St. Jude Children’s Research Hospital, Memphis, TN 38105, USA; Ji-Hoon.Cho@stjude.org (J.-H.C.); Yuxin.Li@stjude.org (Y.L.); Tim.Shaw@stjude.org (T.I.S.); Haiyan.Tan@stjude.org (H.T.); Boer.Xie@stjude.org (B.X.); 2 Department of Structural Biology, St. Jude Children’s Research Hospital, Memphis, TN 38105, USA; Suresh.Poudel@stjude.org (S.P.); Drew.Jones@nyulangone.org (D.R.J.); 3 Department of Developmental Neurobiology, St. Jude Children’s Research Hospital, Memphis, TN 38105, USA; 4 Department of Computational Biology, St. Jude Children’s Research Hospital, Memphis, TN 38105, USA

**Keywords:** metabolomics, metabolome, mass spectrometry, metabolite identification, database search, metabolite formula, metabolite structure, software, algorithm, yeast

## Abstract

Metabolomics is increasingly important for biomedical research, but large-scale metabolite identification in untargeted metabolomics is still challenging. Here, we present Jumbo Mass spectrometry-based Program of Metabolomics (JUMPm) software, a streamlined software tool for identifying potential metabolite formulas and structures in mass spectrometry. During database search, the false discovery rate is evaluated by a target-decoy strategy, where the decoys are produced by breaking the octet rule of chemistry. We illustrated the utility of JUMPm by detecting metabolite formulas and structures from liquid chromatography coupled tandem mass spectrometry (LC-MS/MS) analyses of unlabeled and stable-isotope labeled yeast samples. We also benchmarked the performance of JUMPm by analyzing a mixed sample from a commercially available metabolite library in both hydrophilic and hydrophobic LC-MS/MS. These analyses confirm that metabolite identification can be significantly improved by estimating the element composition in formulas using stable isotope labeling, or by introducing LC retention time during a spectral library search, which are incorporated into JUMPm functions. Finally, we compared the performance of JUMPm and two commonly used programs, Compound Discoverer 3.1 and MZmine 2, with respect to putative metabolite identifications. Our results indicate that JUMPm is an effective tool for metabolite identification of both unlabeled and labeled data in untargeted metabolomics.

## 1. Introduction

Metabolome refers to the complete set of metabolites (or small molecules) in biological samples. In the multi-omics era, the metabolome is regarded as the most downstream stage in the omics spectrum and is highly dynamic, sensitive to molecular changes, and descriptive of the phenotype. Importantly, metabolites also serve as the building blocks to the other macromolecules, and therefore are not just endpoints of the central dogma, but the interface to the environment. Metabolites can function by affecting gene and protein activities through direct interaction and chemical modifications [1]. Large-scale profiling of metabolites can reveal novel mechanisms within a certain biological system. With the advance of analytical technologies and bioinformatics, metabolomics has been routinely applied in biomarker discovery, drug development, nutrition, agriculture, etc. [2,3]. More recently, combined with other omics, metabolomics has been used in basic research, precision health, and disease studies at a comprehensive systems level [4,5]. Liquid chromatography-tandem mass spectrometry (LC-MS/MS)-based untargeted metabolomics has become a powerful method for metabolome profiling, enabling the acquisition of thousands of metabolite features from complex samples [6,7]. Sophisticated informatics tools are required to process large-scale metabolomic data, including feature detection, formula identification, metabolite annotation, false discovery rate (FDR) estimation, and metabolite quantification. However, there are still significant challenges in untargeted metabolomics data processing, which limit the utility of metabolomics in practice and significantly hamper efforts.

Over the past decade, numerous software programs have been developed for untargeted mass spectrometry-based metabolomics, such as XCMS [8], MS-DIAL 4 [9], MZmine 2 [10], SIRIUS 4 [11], and Compound Discoverer (CD, Thermo Scientific), which are either dedicated to individual steps of data processing or designed as a complete workflow to meet the need of different analytical platforms. Among these data processing steps, structural annotation of global data remains technically challenging and has become an active research field [12]. The confidence of metabolite annotation can be classified into four levels [13]. The highest level that the LC-MS/MS approach can achieve is Level 1 in which metabolite structures are identified by in-house authentic standard libraries with a matched MS/MS spectrum, established retention time (RT), and known resolution of related isomers of confounding analytes. When authentic compounds are not available, Level 2 annotation can be reached by matching MS/MS data with experimental databases in the absence of RT matching [14]. More recently, an additional annotation level (i.e., Level 0) has been introduced, requiring a definitive analysis of three-dimensional (3D) structures with pure compounds [15]. A range of publicly and commercially available experimental MS/MS databases and software tools have been developed [15,16]. However, there is limited spectral coverage for the 120+ million known chemical structures deposited in public databases [17], and these collected spectra cannot be simply applied as a universal standard library because the MS/MS spectra are affected by the use of different MS settings and varying instrument performances (resolution and mass accuracy). To alleviate this problem, several in silico fragmentation tools, such as MS-FINDER [18], CFM-ID [19], MetFrag [20], ChemDistiller [21], and CSI:FingerID [22], have been developed to generate theoretical MS/MS databases [15]. Retention time prediction models [23] can facilitate metabolite identification by integrating multiple layers of information to improve confidence, but such predictions are LC method specific and are still exploratory. Moreover, Level 3 allows the identification of tentative metabolite candidates of compound classes, and Level 4 could have metabolite assignments of molecular formulas without defined structures. A list of programs that are commonly used in metabolomics is summarized with pros and cons (Table S1).

With the availability of high-resolution mass spectrometry instruments, the benefits of stable-isotope labeling methods have been recognized including distinguishing endogenous metabolites from background artifacts, increasing the confidence of metabolite identification [24,25], mapping metabolic fates, providing critical insights into pathway dynamics [26], and metabolite quantification [27]. Several programs have been developed for isotope labeled-based analysis [9,26,28,29,30,31], such as feature extraction and small-scale formula annotation, but they are not intended for large-scale metabolite identification.

In this paper, we describe JUMPm (i.e., Jumbo Mass spectrometry-based Program of Metabolomics), a sophisticated and publicly available workflow for large-scale metabolomics data analysis starting from mass spectrometry raw data to metabolite identification. JUMPm is capable of processing stable isotope-labeled (single element labeling or multiple element labeling) and unlabeled LC-MS/MS data. Importantly, we include a false discovery rate (FDR) estimation based on our previously described target-decoy strategy [32] to evaluate the confidence of identified metabolites across a dataset. We further evaluated the performance of JUMPm by comparing it with CD (version 3.1) and MZmine 2 for compound identification.

## 2. Results

### 2.1. Design and Implementation of JUMPm Program

**Figure 1:**
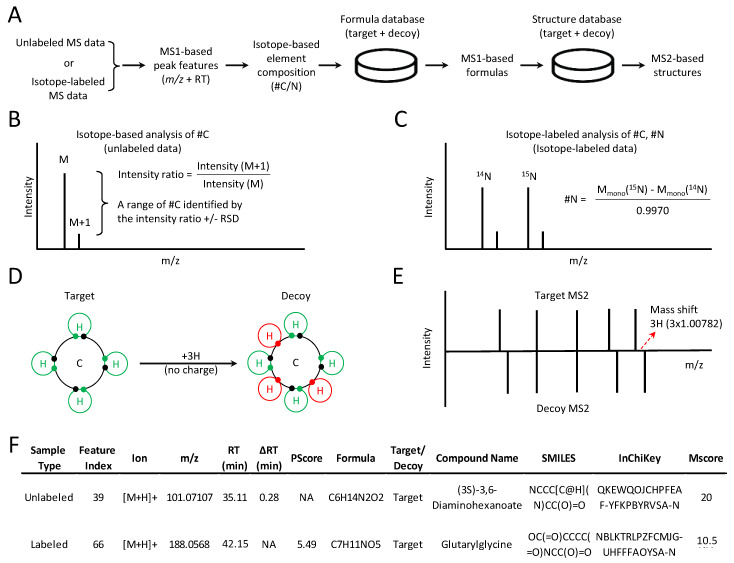
General workflow of Jumbo Mass spectrometry-based Program of Metabolomics (JUMPm) and the target-decoy strategy for FDR estimation. (**A**) JUMPm can identify metabolites in a metabolomic experiment using either unlabeled or labeled data. Inferred stoichiometry is used to limit the search space of formulas. Candidate formulas are used to propose structure identifications which are then scored by MS2; (**B**) The scheme to compute the range of carbon numbers for unlabeled data using natural isotopic distribution. RSD, relative standard deviation; (**C**) The scheme to calculate the nitrogen number using a pair of unlabeled and labeled ions; (**D**) Utilization of the target-decoy strategy. Decoys (invalid structures) are made by violating the octet rule with the addition of three hydrogens without changing the charge state [32]. The impossible decoy structure for methane is shown. The decoy formulas could be incorrectly identified due to chance matches against searched *m/z* values; (**E**) Generation of a decoy MS/MS pattern by mass addition of three hydrogens on a specific atom; (**F**) Examples of JUMPm output for unlabeled and labeled datasets.

We have developed JUMPm, a software tool for large-scale metabolite identification of both unlabeled and stable isotope-labeled data-dependent LC-MS/MS analyses (Figure 1A). The stepwise analysis includes the detection of peak features from MS1 scans, the estimation of element composition (e.g., the number of carbon or nitrogen atoms) in formulas, and the search against formula and structure databases for annotation. If the input is from unlabeled samples, we seek to derive the carbon number in formulas using naturally occurring isotopic distribution (Figure 1B, see details in Materials and Methods). If the input is from stable isotope-labeled samples, JUMPm derives the number of labeled carbon atoms or nitrogen atoms through the accurate mass difference between ion pairs (Figure 1C). This labeling workflow requires individual samples to be analyzed as a mixture of the unlabeled and labeled counterparts. Importantly, we have introduced a simple target-decoy strategy [24] to estimate the FDR in matched formulas and structures. This approach is a widely applied method in MS-based proteomics [33,34] and is implemented, here, in an analogous fashion for large-scale metabolomics. Briefly, decoys are generated by violating the octet rule in chemistry to ensure that the decoy formulas are impossible, and therefore can only be matched by random chance within *m/z* tolerance. For instance, if three hydrogen atoms are added to the target formula of methane to produce a respective decoy formula (Figure 1D), no such structure is possible at the specified charge state. Accordingly, a decoy MS/MS pattern can be generated by adding three hydrogen atoms to the associated atom (Figure 1E) to generate implausible MS/MS ion patterns. When searching against a concatenated database composed of an equal number of targets and decoys, random search values are believed to be equally distributed between the targets and decoys. Thus, FDR can be estimated in authentic searches by examining the proportion of decoy matches, i.e., the number of decoy matches divided by the total number of target matches [24]. Finally, the identified features, formulas, and structures are listed as the output (Figure 1F), and the reliability of structure matches can be judged by MS/MS-associated matching scores (Mscores).

To fully evaluate the JUMPm pipeline, we used LC-MS/MS runs from the following three distinct types of samples (Table 1): (i) reverse phase (RP)-LC-MS/MS of unlabeled metabolites of yeast lysate in positive ion mode, (ii) RP-LC-MS/MS of labeled yeast metabolite lysate in positive ion mode, (iii) LC-MS/MS of a known library of synthetic standards by hydrophilic interaction liquid chromatography (HILIC) in negative ion mode.

**Figure 2:**
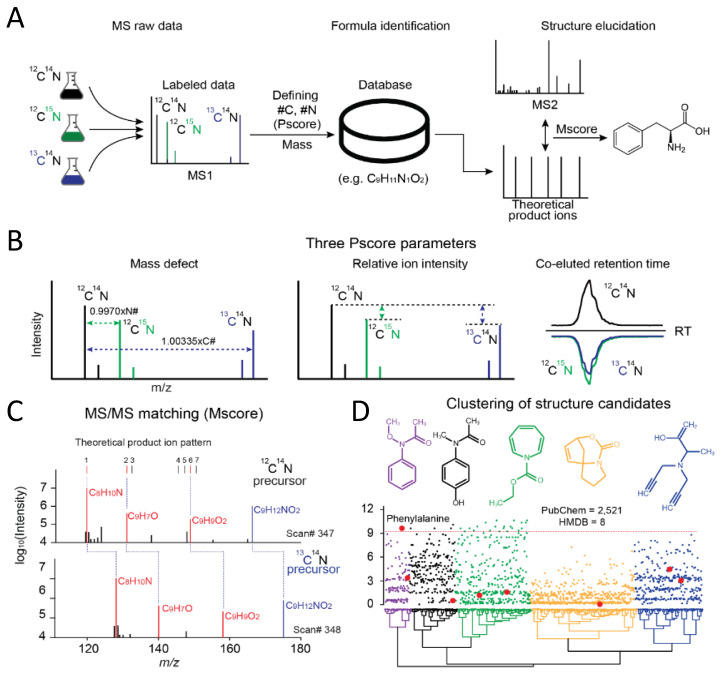
JUMPm analysis of labeled samples and an example identification of phenylalanine. (**A**) Conceptual workflow for a stable isotope-labeled experiment (the double labeled sample is not shown); (**B**) The quality of each isotope-labeled pair is scored with three parameters (mass defect, relative ion intensity, and co-eluted retention time). The Pscore is used to discriminate authentic pairs from random matches; (**C**) For each isotope-labeled pair, the relevant MS2 spectra are scored (Mscore) and annotated with the top match. Three matched theoretical fragment ions are highlighted in red; (**D**) Hierarchical clustering of all structure candidates by predicted product ion intensities for the example metabolite spectrum (HMDB hits, large red dots and PubChem hits, small dots). Representative structures from each colored group are shown. All candidates share the neutral formula C_9_H_11_NO_2_.

Then, we used the complex labeled yeast lysate to illustrate the workflow of JUMPm (Figure S1). The complex lysate consists of four distinct samples (unlabeled, ^13^C-labeled, ^15^N-labeled, and double labeled) that are equally mixed prior to the LC-MS/MS run. JUMPm accepts the LC-MS raw data as input, and then performs deisotoping, noise characterization, and mass calibration to generate a list of peak features (Figures S2 and S3). JUMPm enables precise determination of the formulas’ labeled elements and stoichiometry from the stable isotope-labeled samples. Differentially labeled metabolites are observed in pairing groups of co-eluting ions (Figure 2A). These ion pairs are detected by a pairing score algorithm (Pscore, see details in Materials and Methods) that considers relative isotopic mass differences of the ^13^C and ^15^N labels, relative ion intensity, and co-elution of the putative ion pairs Figure 2B and Figure S4). Finally, the stoichiometry of isotope-labeled elements (C and N) and accurate precursor ion mass identify unique metabolite formulas for a given feature from a theoretical formula database.

Once the metabolite formulas are identified, JUMPm searches the associated MS/MS spectra against a user-defined structure database (Figures S5 and S6), for example, the Human Metabolome Database (HMDB), a public collection of chemical database (PubChem), and the Yeast Metabolome Database (YMDB), to detect structure candidates and rank the candidates by Mscores. The Mscore for each structure candidate is calculated by the hypergeometric test which compares theoretical (*in silico*) MS/MS product ions with the observed MS/MS peaks (Figure 2C and Figure S7). This strategy is also widely used in proteomic pattern-matching analysis [35]. One challenge to this strategy is that a single chemical formula often has many possible structural isomers (37 on average in PubChem) which cannot be differentiated by available MS/MS ions. For example, the formula C_9_H_11_NO_2_ yields 2521 PubChem entries that can be clustered into five structural families, each shown by a representative chemical structure (Figure 2D). In this example, there are 25 isomers with Mscore of at least 9.5, collectively defined as a “metabolite isomer group”. Nevertheless, when the same spectrum is searched against a small biologically focused database (e.g., HMDB), only eight structural isomers are matched (large red dots in Figure 2D), in which only phenylalanine has an Mscore above 9.5 and is ranked as the top candidate. This analysis indicates that excessive search space increases the chance of spurious matches and reduces the possibility of identifying genuine metabolites.

### 2.2. Evaluation of False Discovery Rate Based on the Target-Decoy Strategy by JUMPm

**Figure 3:**
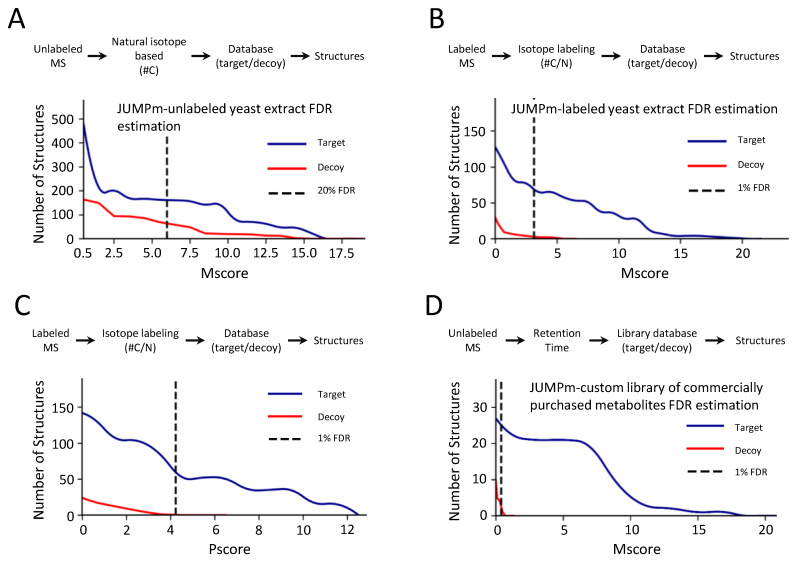
Target-decoy strategy for the estimation of FDR using JUMPm. The x-axis is different JUMP scores (Pscores/Mscores) and the y-axis is the number of structures identified using JUMPm. (**A**) Distributions of target and decoy Mscores from the unlabeled yeast lysate. All matches with zero Mscore are filtered out in the graph; (**B**) Distributions of target and decoy Mscores from the labeled yeast lysate; (**C**) Distributions of target and decoy Pscores from the labeled yeast lysate; (**D**) Distributions of target and decoy Mscores obtained from a custom library search consisting of a mixture of 120 purchased synthetic metabolites. A HILIC column is used to run the cocktail of purchased synthetic metabolites under negative ionization mode.

As FDR is a novel concept implemented in the JUMPm program, we have scrutinized the FDR function using multiple LC-MS/MS runs (Table 1). From the unlabeled yeast run, JUMPm detects both target and decoy metabolite structures with a range of Mscores (Figure 3A). The structure FDR varies at different cutoffs of Mscores. For example, with a stringent cutoff of 6.0, the FDR in the accepted matches is still ~20%, suggesting that a significant level of false assignments cannot be filtered out with this score alone when analyzing the unlabeled data. In sharp contrast, molecular formulas can be precisely determined when using labeled search data. With an Mscore cutoff of just 3.0, the FDR can be reduced to as low as ~1% (Figure 3B). Similarly, the Pscore, an index of ion pairing in the 4-plex sample, can also be used as an additional cutoff to remove false discoveries (Figure 3C). Another strategy for improving the confidence in metabolite identification is to rely on LC retention time. Indeed, from the synthetic standard run on the HILIC column (with known components, Table 1), decoys are almost eliminated when searching against the custom library with defined retention time (Figure 3D). When using the FDR cutoff of ~1%, we identified 119 target components and only 1 decoy hit (real FDR = 1/119 = 0.84%), supporting the application of the target-decoy method. Moreover, the results strongly confirm the notion that metabolite identification can be enhanced by unambiguously defining molecular composition by stable isotope labeling [24], or by using LC retention time in a custom library search.

### 2.3. Performance Comparison with the Other Metabolite Identification Tools

**Figure 4:**
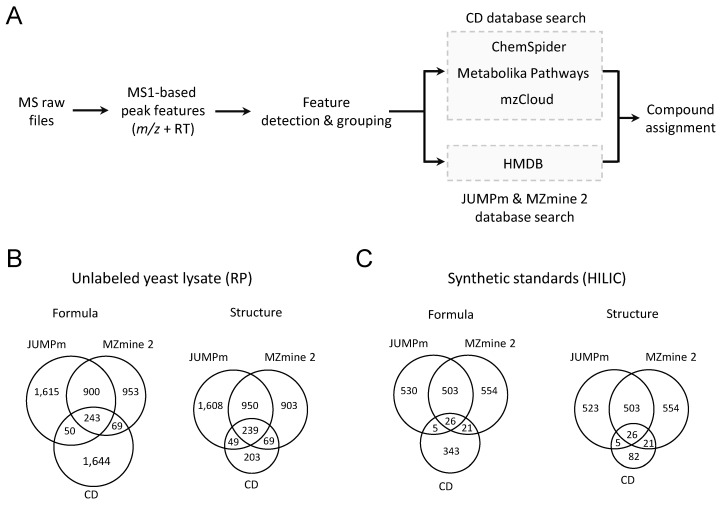
Performance comparison among JUMPm, CD, and MZmine 2 on two LC-MS/MS runs, i.e., the unlabeled yeast lysate and a mixture of synthetic standards. (**A**) The workflow of CD and MZmine 2 (Figure 2) uses the HMDB database; (**B**) Number of formulas and structures detected from the sample of unlabeled yeast lysate by the three software tools. RP, reversed phase; (**C**) Number of formulas and structures detected from the sample of synthetic standards. HILIC. hydrophilic interaction liquid chromatography.

To further evaluate the performance of JUMPm, we compared the results of metabolite identification among JUMPm, CD (version 3.1), and MZmine 2 (version 2.53). Both CD and MZmine 2 are two widely used programs available for untargeted metabolomics. In CD and MZmine 2, the raw MS files are first used to extract peak features. Then, the peak features and associated MS/MS scans are utilized to search the databases to assign formulas and compound structures (Figure 4A). Using unlabeled yeast lysate 2827, 2006, and 2165 formulas are detected by JUMPm, CD, and MZmine 2, corresponding to 2846, 560, and 2161 structures, respectively (Figure 4B). CD identifies slightly fewer but comparable formulas, with a significant reduction in the identification of structures. One possible explanation is that CD uses the mzLogic algorithm, which combines identifications from ChemSpider and Metabolika Pathways with the spectral library information from the mzCloud online. Alternatively, both JUMPm and MZmine 2 use the HMDB, a larger database than the spectral library in CD. A comparison of the structural identification for JUMPm and MZmine 2 shows that about 55% (1189/2161) structures detected by MZmine 2 can be identified by JUMPm, whereas 41% (1189/2846) identifications from JUMPm can be detected by MZmine 2. The difference in identification from JUMPm and MZmine 2 could be attributed to the difference in data preprocessing, feature extraction, and scoring algorithm. In addition, we have conducted a comparison of the three tools using a mixture of synthetic standards with similar results (Figure 4C). It should be mentioned that the FDR analysis is not available in CD or MZmine 2, and therefore FDR filtering function is not applied in JUMPm during this comparison.

## 3. Discussion

We have developed the JUMPm software package for automated processing of large-scale metabolomics datasets. JUMPm is capable of analyzing fully stable isotope-labeled, and unlabeled MS data for confident formula identification and structural interpretation. The program also allows for the use of theoretical and experimental databases, as well as a spectral library with RT information. Moreover, different types of LC-MS/MS runs are used to test the JUMPm program. During the analysis of the unlabeled and labeled yeast samples, we have recognized the challenges of defining reliable metabolites, from MS raw data to peak features, possible formulas, and putative structures. The assignment of formulas to peak features can be enhanced by estimating carbon composition from the carbon-associated isotopic distributions, or by precise determination of element composition through fully stable isotope labeling [36]. The transition from formulas to structures is also complicated by the presence of a large number of structural isomers, which can be alleviated by the introduction of synthetic standards with RT information and known MS/MS patterns. Finally, the computer-derived output should still be examined with caution.

Furthermore, we have implemented the newly developed target-decoy strategy to evaluate the FDR [32]. The strategy is useful to estimate the degree of random MS/MS assignments based on a null hypothesis, in which the decoys should be equal to the targets and share similar physical properties to the targets. Adding a small odd number of hydrogen atoms (e.g., one, three, or five) to the targets violates the octet rule and fits the requirements of generating the decoys [32]. Three-hydrogen addition is selected in this method, because one-hydrogen addition can generate isobaric ions to the naturally occurring isotopic ions, and five-hydrogen addition leads to a larger mass shift. Using the synthetic library sample, the estimated FDR is highly consistent with the real FDR, demonstrating the validity of the target-decoy method. Finally, the source code of JUMPm is publicly available, with a modular design and implementation that easily enables new algorithms to be added. Thus, we believe that JUMPm is a complementary tool for metabolite identification in the studies of untargeted metabolomics.

## 4. Materials and Methods 

### 4.1. Reagents

The reagents included the following: LC-MS grade acetonitrile (ACN); water; ammonium acetate; ammonium sulfate; ammonium hydroxide; and formic acid (Sigma-Aldrich, St. Louis, MD, USA); 0.5 mm disruption beads (RPI); Difco^TM^ yeast nitrogen base (BD Bioscience, San Jose, CA, USA); dextrose (Fisher Bioreagents, Fair Lawn, NJ, USA); ^13^C-6 glucose; ^15^N-2 ammonium sulfate (Cambridge Isotope Laboratories, Tewksbury, MA, USA); Mass Spectrometry Metabolite Library of Standards (IROA Technologies, Sea Girt, NJ, USA); 1.9 µm C18 beads (Dr. Maisch GmbH, Germany); 75 µm ID empty silica column (New Objective, Woburn, MA, USA); and SeQuant ZIC-HILIC column (MilliporeSigma, Burlington, VT, USA).

### 4.2. Isotope Labeling Protocol

*S. cerevisiae* (Fleischmann) cells were grown in four different minimal media conditions. A control media consisted of the following natural isotopic abundance components: Difco^TM^ yeast nitrogen base without amino acids and ammonium sulfate (BD Biosciences), with 5 g/L ammonium sulfate (Sigma) and 20 g/L glucose (Sigma). For carbon-13 labeling, the media remained the same, except that ^13^C-6 glucose (Cambridge Isotope Laboratories) was used in place of standard glucose. Similarly, for nitrogen-15 labeling, ^15^N-2 ammonium sulfate was substituted into the media. Each culture was maintained for ~30 generations in the labeled media before analysis. Cultures were seeded to an OD_600_ of 0.1 and allowed to grow to 1 (~6 generations).

### 4.3. Sample Preparation

Each sample was harvested, and then subjected to a universal metabolite extraction procedure. The liquid culture was transferred to 15 mL conical vials and centrifuged at 1000× *g* for 3 min to obtain a cell pellet. The supernatant was discarded and 1 mL of freezing 80% acetonitrile was added along with disruption beads to facilitate lysis. Each vial was subjected to 3 min of vortexing at 3000 rpm in a 1 on/1 off pattern to maintain sample temperature [6]. The lysate was transferred to a fresh vial to exclude the beads, and then centrifuged at 21,000× *g* for 5 min to clarify the liquid phase. Metabolite concentrations in the lysates were measured using UV absorbance at 300 nm, and then equally pooled together as a 4-plex sample. The pooled mixture was aliquoted, speed vacuum dried, and stored at −80 °C until LC-MS analysis. Metabolite Library of Standards (IROA Technologies) were prepared according to the manufacturer’s instruction. 

### 4.4. LC-MS Analysis and Parameters

All samples were analyzed on an Orbitrap Q Exactive HF (Thermo Scientific) coupled to Waters nanoAcquity UPLC using a previously optimized protocol [6,36]. The chromatographic stationary phase was a nanoscale column (75 μm × 100 mm) packed with 1.9 μm C18 beads to facilitate the reverse-phase analysis or a SeQuant ZIC-HILIC column (2.1 mm × 150 mm, 3.5 μm resin) for chromatographic separation of polar metabolites. For the reverse phase C18 separation, mobile phases A and B each consisted of LC-MS grade water or acetonitrile, respectively, with 0.2% formic acid. The sample injection volume was 2 μL, while the system flow rate was 0.25 μL/min with a biphasic gradient in 60 min. For the HILIC analysis, 10 mM ammonium acetate in LC-MS grade water or acetonitrile with pH 8 was used for mobile phase A and B. The sample injection volume was 2 µL, while the system flow rate was 0.1 mL/min with a 90 min triphasic gradient. Full MS scans used a resolution of 120,000 from *m/z* 100–1000. Automatic gain control was set to 3e6 for full MS. A top 20 MS/MS method used HCD at a stepped normalized collision energy of 50, 100, and 150 for fragmentation and a resolution of 30,000 in the Orbitrap. Isolation width in the ion trap was 1 Da. Each MS/MS scan had a target of 3e5 automatic gain control. Dynamic exclusion was set at 20 s.

### 4.5. Construction of the Theoretical Mass-Formula Database

To quickly retrieve possible formulas for a given mass value, we generated a mass-formula database containing all possible formulas and their corresponding theoretical neutral monoisotopic masses. The composition of elements included carbon (C), hydrogen (H), nitrogen (N), oxygen (O), phosphorus (P), and sulfur (S), with default ranges of C[0–105], H[0–170], N[0–30], O[0–40], P[0–4], and S[0–4] in the Human Metabolome Database (HMDB). The theoretical formulas were filtered by the ring double bond equivalents (RDBE) rule. The maximum mass was set to 1500 Da with ~265 million entries, indexed by mass and element composition.

### 4.6. Feature Detection and Signal-to-Noise Definition

The raw LC-MS data (.raw format) were converted into .mzXML format using ReAdW (http://tools.proteomecenter.org). This program extracts ion features in an MS scan using the method reported by Cox and Mann [37]. Peaks, in an MS scan, were detected by fitting data in profile mode to a Gaussian distribution (referred to as two-dimensional (2D) peaks of *m/z* and intensity), and then assembled into three-dimensional (3D) peaks (*m/z*, intensity, and retention time) as metabolite features. Seven parameters could be adjusted, such as (i) minimum 2D intensity, (ii) *m/z* tolerance, (iii) the minimum number of consecutive MS1 scans for defining 3D peaks. All 2D and 3D peaks were saved in binary files for subsequent analyses.

In complex samples, feature extraction is complicated by a mixture of signal and noise peaks. The noise peaks may be derived from electrical and chemical sources, and are mostly not reproducible from scans to scans in LC-MS/MS. Thus, we defined candidate “noise” peaks as those that could not be replicated in adjacent MS1 scans. The noise level was defined as the mean of the intensities of all noise peaks for each scan after outlier removal. We filtered metabolite features with a cutoff of 3 signal-to-noise (S/N) ratio.

### 4.7. Mass Calibration

Mass accuracy is a crucial parameter for determining the metabolite formula and generating confident annotations. JUMPm is able to calibrate raw *m/z* values based on a known internal standard generated from electrospray of air, polydimethyl-cyclosiloxane ([Si(CH_3_)_2_O))_6_ + H]^+^, *m/z* 445.120025) [38], which is present in most scans. We divided the entire elution profile into 10 windows and calibrated in each window. If the number of detected polysiloxane ions is less than 5% of all scans, the program does not perform this function. After mass calibration, we used a mass tolerance as small as 4 ppm (default) to extract metabolite features followed by deisotoping and peak pairing.

### 4.8. Detection and Scoring of Candidate Peak Pairs

Candidate metabolite features are selected by pairing ^12^C^14^N, ^12^C^15^N and ^13^C^14^N peaks with mass defects (e.g., $D_{n,c}$ = 0.99703 for ^15^N, and $D_{c,c}$ = 1.00335 for ^13^C):

$$D_{n,c}=\frac{M\left(N15\right)-M\left(C12\right)}{||M\left(N15\right)-M(C12)||}\leq 0.99703\pm \mathrm{tolerance}$$

where “*M*” represents the mass of a peak, and “|| ||” represents the ceiling of the mass difference.

To discriminate authentic pairs from by-chance matches, JUMPm generates a combined Pscore based on the mass defect, relative ion intensity, and co-elution of the putative ion pairs. Under similar conditions, the unlabeled and labeled metabolites are expected to show comparable intensities in the ^12^C-^13^C and ^12^C-^15^N pairs.

#### 4.8.1. Calculating *p* Value for Mass Defect

JUMPm generates a *p* value to determine the mass defect for the ^12^C-^13^C and ^12^C-^15^N candidate pairs. To calculating the *p* value, we assume that the random shift of each peak follows the normal distribution:

$$M\sim N\left(\mu ,\;\sigma^{2}\right)$$

where μ and σ represent mean and standard deviation of the peak, respectively.

The mass defect can be calculated as follows:

$$D_{m}=\frac{M\left(N\right)-M\left(C12\right)}{||M\left(N\right)-M\left(C12\right)||}$$

thus, the $D_{m}$ is normal distributed because the numerator (i.e., M(N)−M(C12)) follows a normal distribution and denominator (i.e., ||M(N)−M(C12)||) is an integer value,

$$D_{m}\sim N\left(\mu ,\;\sigma^{2}\right)$$

where μ = 0.997035 for N mass defect and μ = 1.003355 for C mass defect; the scale parameter $\sigma^{2}$ can be estimated from authentic peak pairs, or targets (defined below) in our case.

#### 4.8.2. Calculating *p* Value for Relative Intensity

JUMPm generates a *p* value to determine the relative intensity for candidate pairs. We assume that the signal intensity of the peaks follows an exponential distribution, and thus log_2_(Intensity) follows a normal distribution as below,

$$log_{2}\left(I\right)\sim N\left(\mu ,\;\sigma^{2}\right)$$

thus, the logarithm of the intensity ratio between ^12^C-^13^C and ^12^C-^15^N peaks follows a normal distribution with the mean μ and the standard deviation σ,

$$log_{2}\left(\frac{I_{x}}{I_{y}}\right)=log_{2}\left(I_{x}\right)-log_{2}\left(I_{y}\right)\sim N\left(\mu ,\;\sigma^{2}\right)\;$$

#### 4.8.3. Calculating *p* Value for Pearson Correlation (Co-Elution)

JUMPm generates a *p* value to assess how well the paired peaks correlate in intensity over co-elution time. The *p* value is calculated based on the relative intensities of each pair using Pearson correlation and *t* statistic with *n* − 2 degrees of freedom:

$$\frac{r\sqrt{n-2}}{\sqrt{1-r^{2}}}\sim t\left(n-2\right)$$

where *r* is the correlation coefficient, and *n* is the number of scans across co-elution time.

#### 4.8.4. Generating the Combined Pair Score

Finally, JUMPm uses Fisher’s method to generate a combined pair score (i.e., Pscore). All six *p* values (two pairs, ^12^C-^13^C and ^12^C-^15^N, for each test) are combined using the Chi-square test,

$$\chi_{2k}^{2}=-2\overset{k}{\underset{i=1}{\sum }}\ln\left(p_{i}\right)$$

where $p_{i}$ represents the *p* value for the *i*th of the above tests, and *k* represents the number of *p* values used for combination (i.e., 6 for our case). The Pscore is calculated as follows:

Pscore=−log(p value)

### 4.9. Formula Identification

JUMPm searches for candidate formula(s) based on the accurate precursor mass and the specified number of carbon and nitrogen atoms (determined from the mass shift of the isotope-labeled pairs). We used the following two limiting criteria for the search: (i) narrowing the search within a user-defined mass tolerance (e.g., 10 ppm) and (ii) matching the required elemental composition. For the example in Figure 2A, the monoisotopic unlabeled peak is *m/z* 166.0856, so JUMPm finds the surrounding 10 ppm indexed segments of the mass-formula database. From the mass shifts of the isotope labels (*m/z* 175.1156 and *m/z* 167.0826 for carbon-13 and nitrogen-15, respectively), JUMPm determines that this formula must have exactly 9 carbons and 1 nitrogen. Only one formula (C_9_H_11_NO_2_) fits these criteria within the lookup range. JUMPm can process both positive and negative mode LC-MS data, with the polarity being a user-selectable option in the parameter file.

### 4.10. Structure Database and Identification

To generate a structure database, we used three databases including PubChem, HMDB, and YMDB. The PubChem database in XML format was downloaded from the FTP (ftp://ftp.ncbi.nlm.nih.gov/pubchem/). We parsed the XML file and extracted the metadata for each entry from the database using an in-house script. The extracted metadata included PubChem ID, formula, InChI key, InChI string, SMILES, IUPAC, and monoisotopic mass for each structure. The database was indexed into the same two layers (i.e., mass and elemental composition) as the theoretical mass-formula database. The Human Metabolome Database (HMDB3.0; http://www.hmdb.ca) and the Yeast Metabolome Database (YMDB1.0; http://www.ymdb.ca) were processed as described for PubChem. The user can choose any of these default databases for analysis. JUMPm can also use any custom database of structures as long as it matches the same generic format described above.

After identifying the corresponding formula(s) for each peak pair, JUMPm searches for all potential structures in the local structure database based on its formula. If a candidate structure is present in the database, JUMPm retrieves its SMILES, PubChem ID, InChI key, and IUPAC name. The SMILES is used for subsequent in silico fragmentation and MS2 scoring.

JUMPm uses MetFrag [39], a predictive fragmentation program to generate MS/MS theoretical product ions. JUMPm also ensures that the mass and formula of the candidate are the same as the precursor of the MS2 scan. If the MS2 spectrum derives from ^15^N or ^13^C instead of a ^12^C precursor, then the predicted MS/MS ions are adjusted to include these heavy isotopes. In addition, users can choose CFM-ID [40], an alternative predictive fragmentation program in JUMPm.

### 4.11. Structure Scoring (Mscore)

JUMPm uses the hypergeometric test to compare theoretical fragments generated by either MetFrag or CFM-ID program to the measured MS/MS ions:

$$p\;value=\frac{\left(\begin{array}{c} n_{1}\\ m \end{array}\right)\left(\begin{array}{c} n-n_{1}\\ k-m \end{array}\right)}{\left(\begin{array}{c} n\\ k \end{array}\right)}$$

where *n* is all possible ion locations, calculated as the mass range of the MS2 spectrum divided by the mass tolerance; *k* is the total number of theoretical product ions of the metabolite structure; *n*1 is the number of detected product ions within the MS2 mass range; and *m* is the number of matched product ions for each structure. On the basis of the *p* value from the hypergeometric test, JUMPm generates an Mscore as below:

Mscore=−log(p value)

### 4.12. Structure Clustering

We performed structure clustering based on theoretical product ions for candidate structures. A binary matrix table was constructed representing the presence or absence of fragmented chemical formulas for each structure, and a distance matrix was calculated using the Jaccard similarity coefficient. To distinguish between groups with similar structures, hierarchical clustering was performed via Ward’s method [41] followed by careful manual inspection. Then, a representative structure for each cluster was determined by the following five steps: (i) for each fragment chemical formula, the percentage of structures containing that formula was identified; (ii) for all formulas present in the fragmented structure, their percentage was derived from Step 1; (iii) for all formulas absent in the fragmented structure, one minus their decimal percentage derived from step 1 was summed together; (iv) for each structure, the values derived from Steps 2 and 3 were summed together; (v) The structure with the highest score was selected as the cluster’s representative structure.

For Compound Discoverer, 10 ppm for *m/z* tolerance, and 10 for noise threshold were used. The remaining parameters were set as the defaults.

### 4.13. Input and Output

JUMPm can take either .raw (Thermo^TM^) files or .mzXML files as input. JUMPm can simultaneously search multiple raw files with the same parameter settings, and it also allows the user to specify certain scan regions for search. The output includes four tables in flat text format. The first (feature table) is a table containing all detected features, including mass values (*m/z*), intensity, and signal vs. noise ratio. The second (formula table) is an exhaustive list of all detected formulas and reports the observed mass values (*m/z*) for the three labels (i.e., ^12^C^14^N, ^12^C^15^N, and ^13^C^14^N) for each peak pair, the pair score, retention time, relative intensity, and chemical formula for each pair. The third (spectrum matching table) contains all candidate structures for each peak pair and their MS2 matching score in addition to the mass and formula in the formula file. The fourth (structure table) lists the top structure (with the highest matching score) for each pair in the spectrum matching table.

### 4.14. Parallel Computing and High-Performance Computation

JUMPm is designed for a parallel computing system, which can significantly increase the processing speed. For our current system, we executed JUMPm on a cluster of 512 cores, 2.3 GHz, 128 GB ram. For the LC-MS yeast metabolite analysis, the .raw file with ~18,000 scans required about 0.5 h to analyze, however, the analyses of two independent .raw files did not take 2X the computational time, because of the parallel nature of the process.

### 4.15. Parameters Used in CD and MZmine 2

For CD, a mass tolerance of 10 ppm was used for both noise filtering and database search, and default values were set for other parameters. For MZmine 2, Automated Data Analysis Pipeline (ADAP) was used for data pre-processing, including chromatogram builder, and wavelet deconvolution (MS2 mass tolerance of 0.01 Da; RT range of 0.2 min). Features were detected by the GridMass algorithm, followed by peak deisotoping and alignment using the random sample consensus (RANSAC) algorithm.

### 4.16. Software

JUMPm is programmed in Perl, JAVA, and R, and can be executed on a Linux cluster system. Analytical variables are controlled with a text-based parameter (.param) file which can be edited by the user to meet their specific needs. The source code and raw data can be downloaded at http://www.stjuderesearch.org/site/lab/peng/jumpm.

## Figures and Tables

**Table 1:** LC-MS/MS runs of metabolites used in this study.

Sample Name	Sample Introduction	LC	MS Ionization Mode
Unlabeled yeast lysate	Unlabeled yeast sample	RP	Positive
Labeled yeast lysate	4-plex mixture of one unlabeled sample and three stable-isotope-labeled yeast samples (C13, N15, and double labeling)	RP	Positive
Synthetic standards (HILIC)	A mixture of purchased synthetic metabolites	HILIC	Negative

Yeast extracts and a synthetic standard mix were analyzed by LC-MS/MS with a reverse phase (RP) or HILIC column in positive or negative ion mode.

## Supplementary Materials

The following are available online at https://www.mdpi.com/2218-1989/10/5/190/s1. Figure S1: Detailed workflow for JUMPm, Figure S2: Distribution of signal and noise levels in the testing dataset, Figure S3: Mass calibration by moving average method, Figure S4: Statistical distribution used for pair scoring, Figure S5: Comparison of the PubChem, Human Metabolome Database (HMDB), and Yeast Metabolome Database (YMDB) databases, Figure S6: Number of molecular formulas containing 16 selected elements in HMDB and YMDB, Figure S7: Scoring of metabolite-spectrum matches, Table S1: List of common software tools in metabolomics.
