# Supplementary material for: JUMPm: A Tool for Large-Scale Identification of Metabolites in Untargeted Metabolomics
Source: Metabolites. 2020 May 12;10(5):190. doi: 10.3390/metabo10050190 (PMC7281133; doi:10.3390/metabo10050190)
Supplement: Supplementary file 1 [file metabolites-10-00190-s001.pdf]

Supporting Information for
JUMPm: a Tool for Large-scale Identification of Metabolites in Untargeted Metabolomics

Xusheng Wang [1, 6, 8, *], Ji-Hoon Cho [1, 8], Suresh Poudel [2, 3, 8], Yuxin Li [1, 2, 3], Drew R. Jones [2, 3, 7], Timothy I. Shaw [1, 4], Haiyan Tan [1], Boer Xie [1, 2, 3], and Junmin Peng [1, 2, 3, *]

[1] Center for Proteomics and Metabolomics, St. Jude Children's Research Hospital, Memphis, TN 38105, USA
[2] Department of Structural Biology, St. Jude Children's Research Hospital, Memphis, TN 38105, USA
[3] Department of Developmental Neurobiology, St. Jude Children's Research Hospital, Memphis, TN 38105, USA
[4] Department of Computational Biology, St. Jude Children's Research Hospital, Memphis, TN 38105, USA
[6] Current address: Department of Biology, University of North Dakota, Grand Forks, ND 58202, USA
[7] Current address: Department of Biochemistry and Molecular Pharmacology, New York University School of Medicine, New York, NY 10016, USA
[8] These authors contributed equally to this work
* Correspondence: xusheng.wang@UND.edu, Tel: 701-777-4673; junmin.peng@stjude.org, Tel.: 901-595-7499

Table of content

Figure S1. Detailed workflow for JUMPm. 3
Figure S2. Distribution of signal and noise levels in the testing dataset. 4
Figure S3. Mass calibration by moving average method. 5
Figure S4. Statistical distribution used for pair scoring. 6
Figure S5. Comparison of the PubChem, Human Metabolome Database (HMDB), and Yeast Metabolome Database (YMDB) databases. 7
Figure S6. Number of molecular formulas containing 16 selected elements in HMDB and YMDB. 8
Figure S7. Scoring of metabolite-spectrum matches. 9

Table S1. List of software tools used in metabolomics data analysis. 10

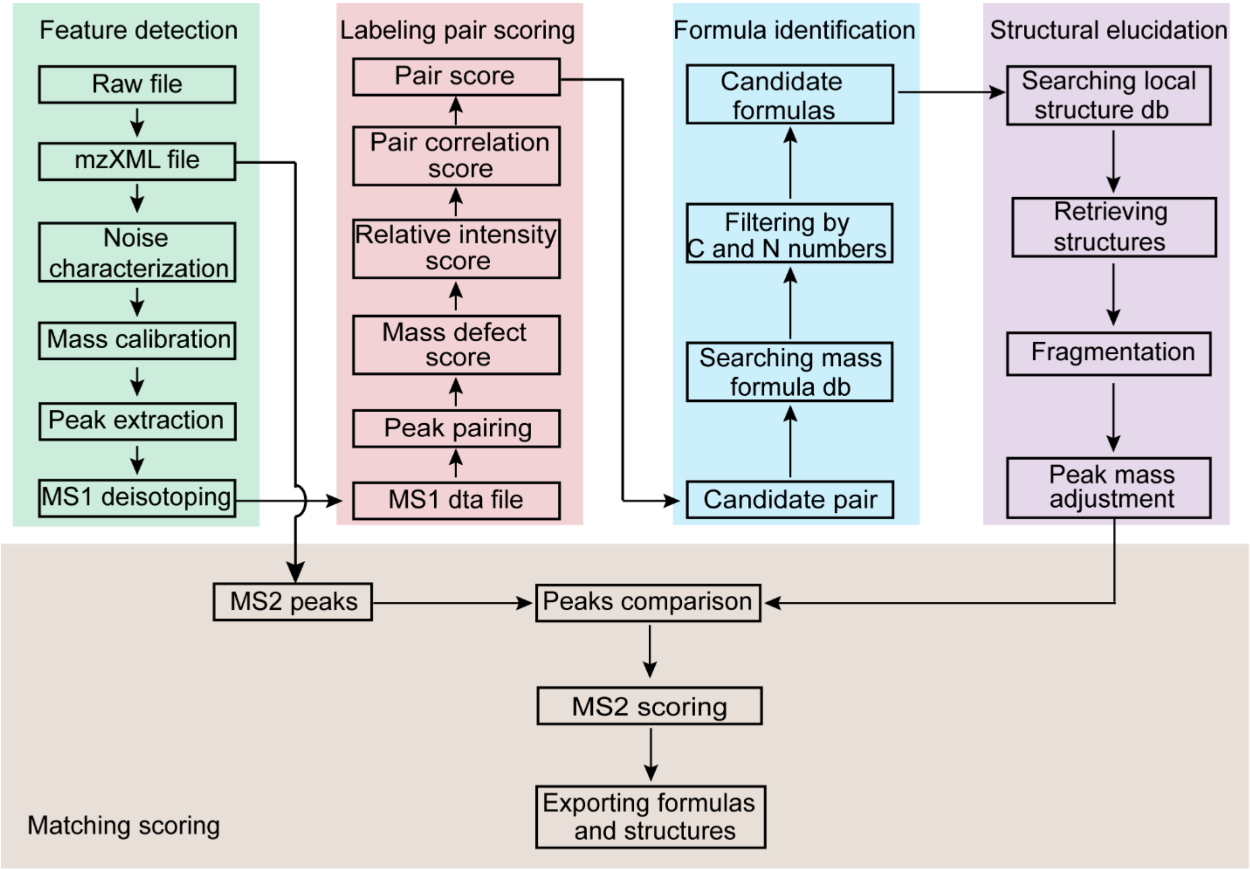

Figure S1. Detailed workflow for JUMPm. JUMPm has five major components, including feature detection, isotope peak pairing, formula identification, structural elucidation, and match scoring. JUMPm accepts .raw or .mzXML files (including MS1 and MS2) and outputs tables of identified metabolites and formulas.

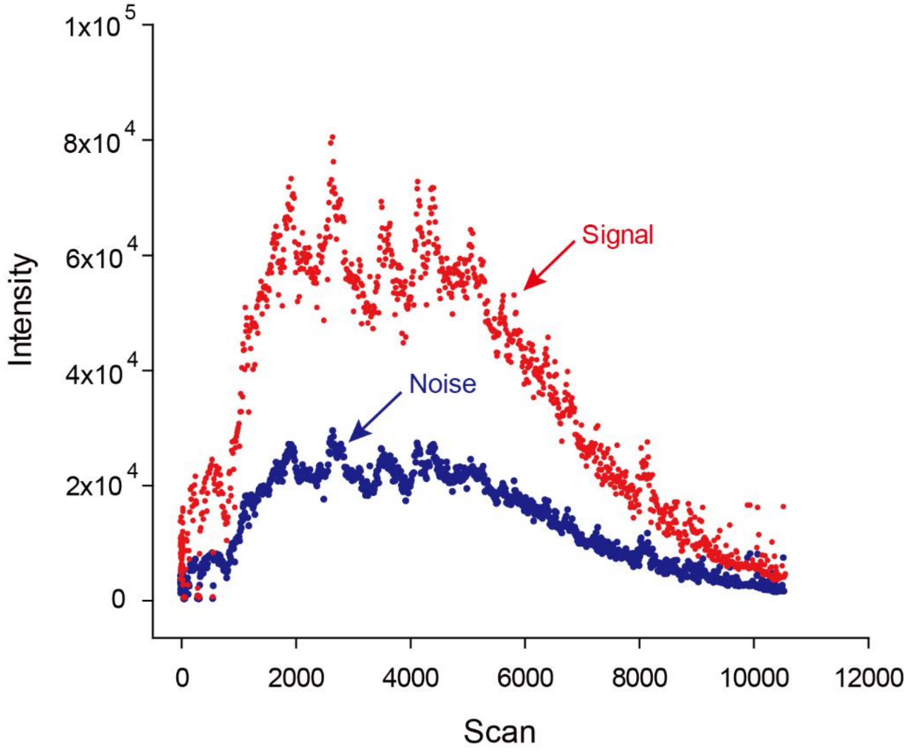

Figure S2. Distribution of signal and noise levels in the testing dataset. JUMPm defines noise peaks as those which cannot be repeatedly detected in adjacent scans (i.e. peaks detected only in a single scan). For each scan, the program collects these noise peaks, removes outliers and obtains the average intensity of remaining peaks. The average is used as scan-specific noise level to enable the calculation of signal-to-noise (S/N) ratio of all peaks. The S/N ratio of 3 is set as the default cutoff for the peaks.

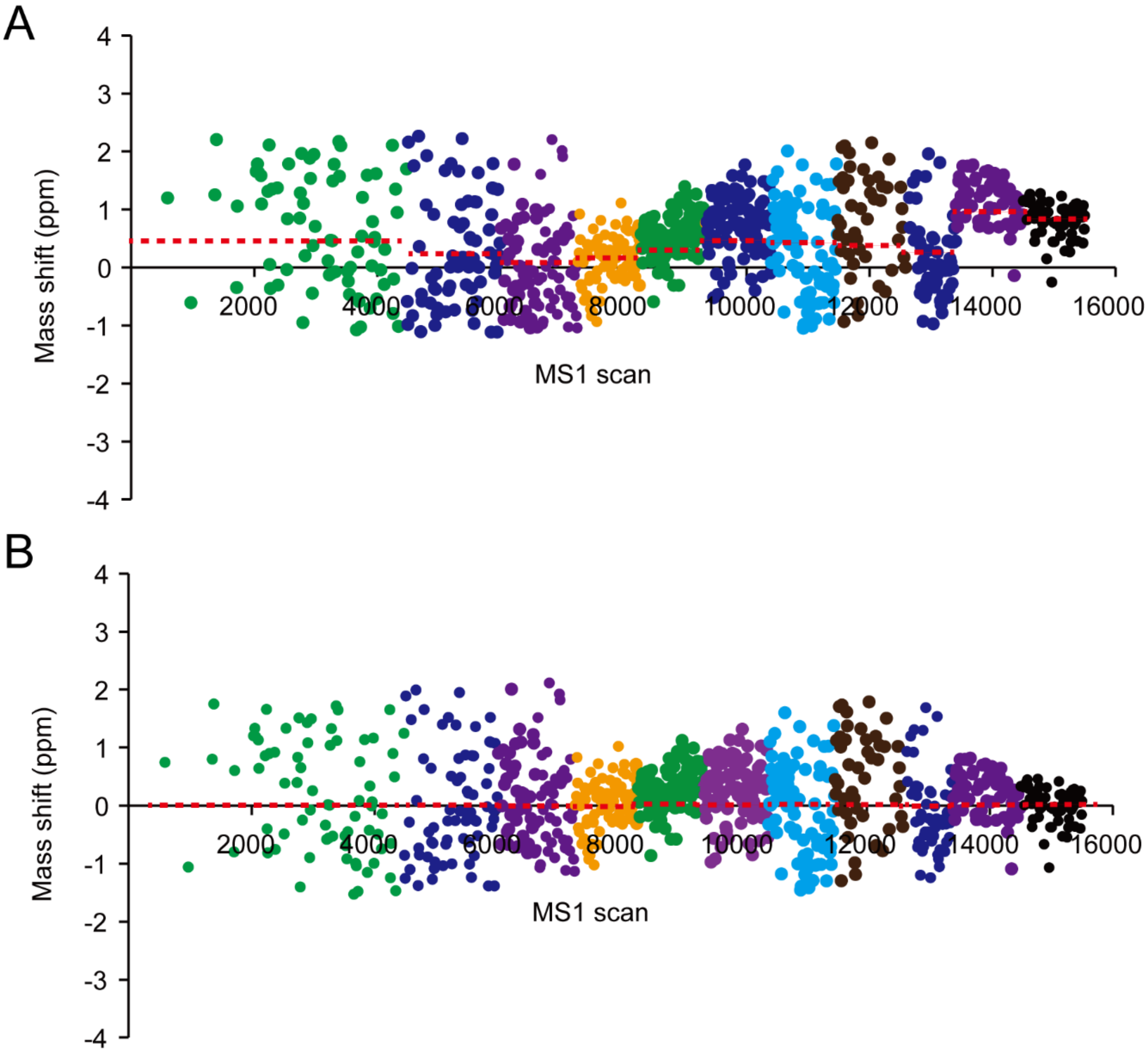

Figure S3. Mass calibration by moving average method. (**A**) Before calibration (**B**) After calibration. Mass accuracy can drift during the course of a single run, and therefore the use of moving windows enables accurate mass correction. The mass error is determined by the mass difference between the detected and theoretical mass of the polysiloxane ion (445.120025). The entire chromatogram is typically divided into 10 windows, each containing the same number of scans. The mean of the mass errors in each window (dashed red line) is used as the calibration value.

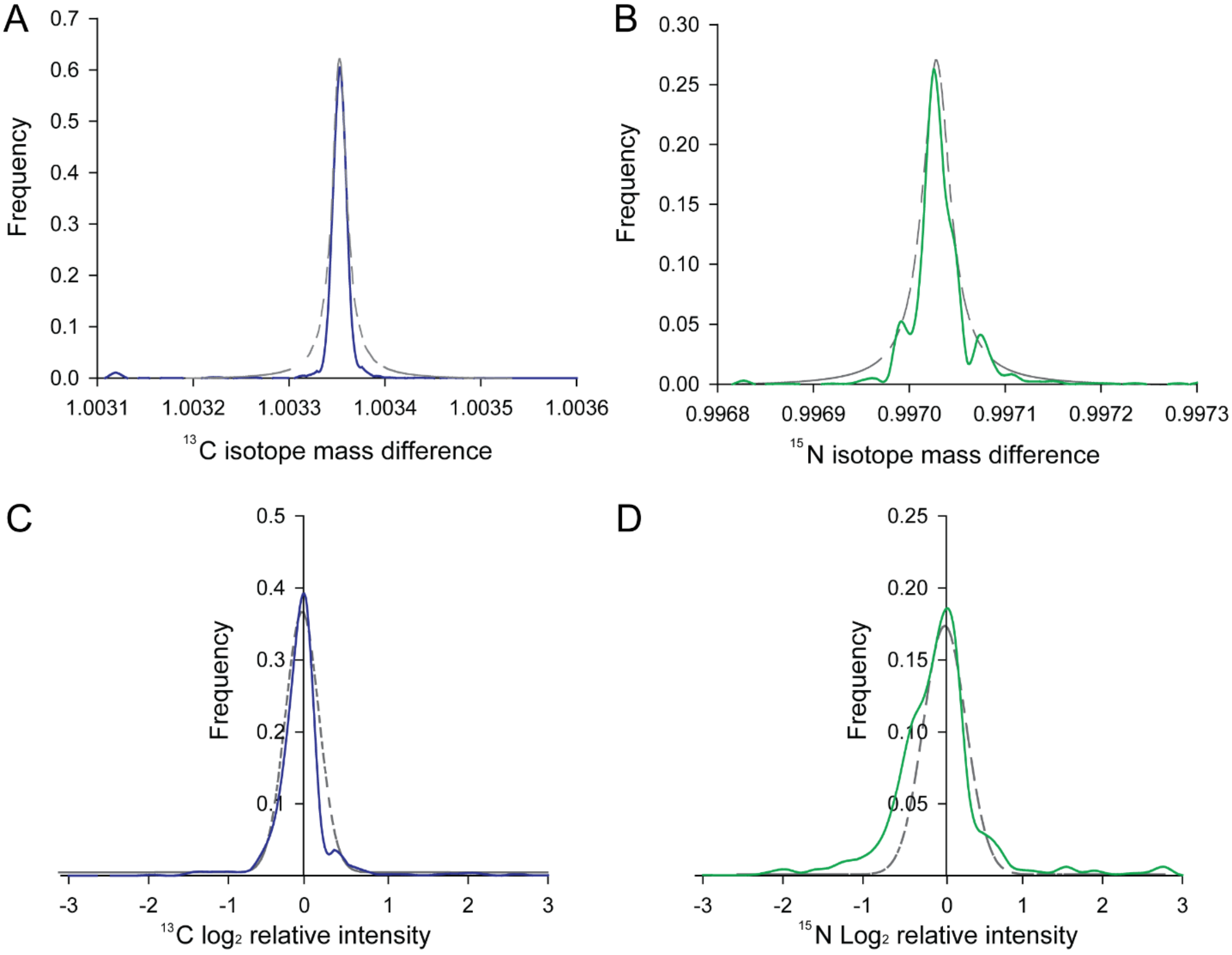

Figure S4. Statistical distribution used for pair scoring. Theoretical distribution (dash line) and frequency histogram (solid line) for isotope mass difference (**A-B**) and relative intensity of the labeled peaks (**C-D**). Both the isotope mass difference and $\log_2$ relative intensity follow the Gaussian distribution, respectively. The parameters associated with the theoretical distributions are estimated from the data. For each pair, JUMPm calculates two *p* values for carbon and nitrogen isotope mass differences and two *p* values for $\log_2$ relative intensity with normal distribution. A final pair score is defined by combining the *p* values from mass difference, relative intensity as well as peak correlation using Chi-square test.

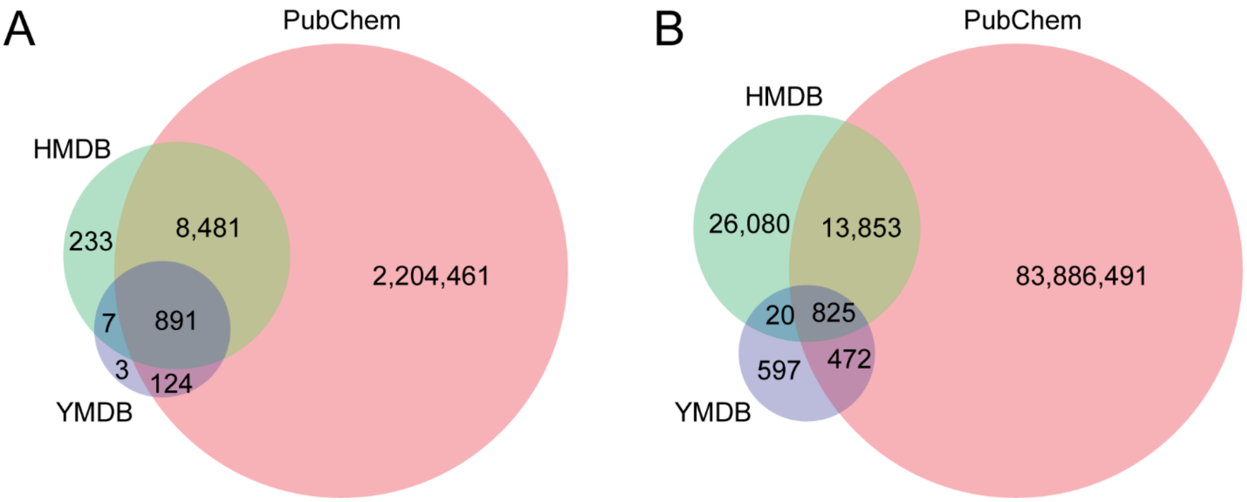

Figure S5. Comparison of the PubChem, Human Metabolome Database (HMDB), and Yeast Metabolome Database (YMDB) databases. We downloaded local copies of these publically available compound/metabolite databases to determine the degree of redundancy between data sources. (**A**) Formulas, (**B**) Structures.

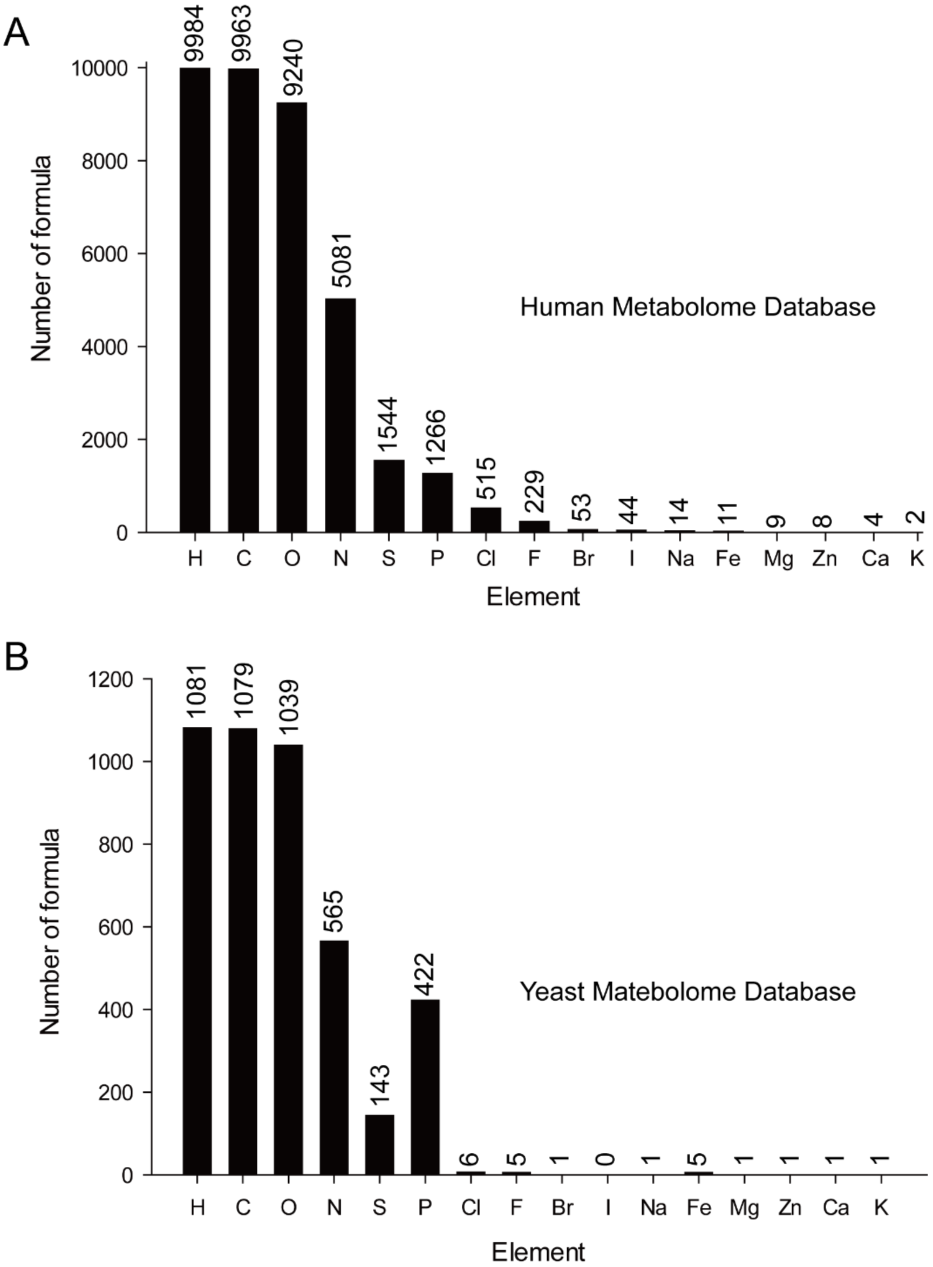

Figure S6. Number of molecular formulas containing 16 selected elements in HMDB and YMDB. We aimed to determine the relative representation of 16 common biological elements across the three metabolite databases used in JUMPm. Outside of carbon, hydrogen, nitrogen, oxygen, phosphorus, and sulfur, the remaining elements were uncommon.

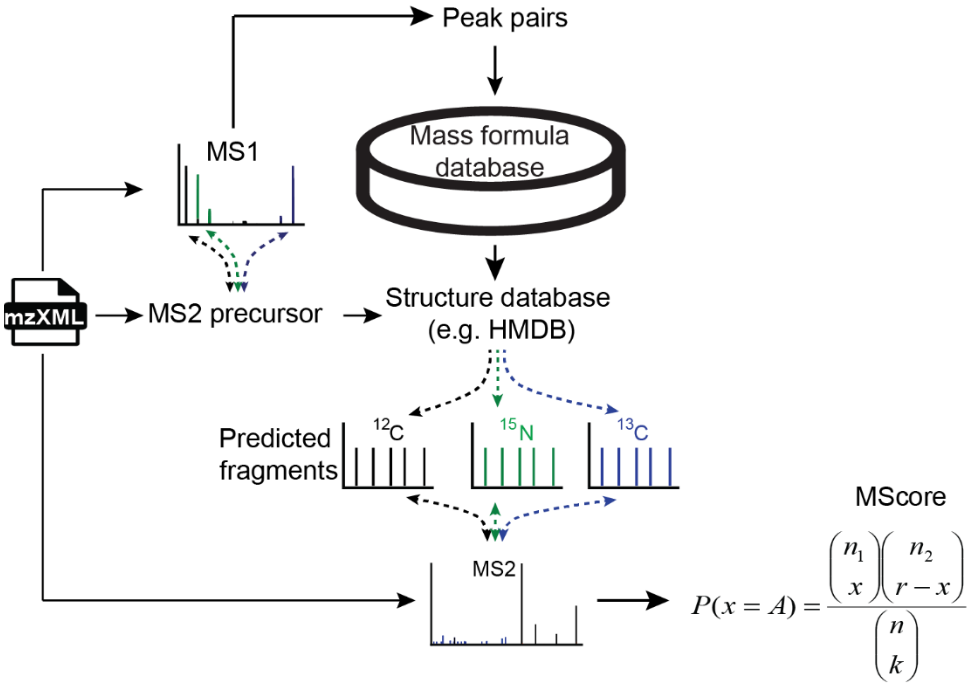

Figure S7. Scoring of metabolite-spectrum matches. Putative formulas are searched against the structure database to detect metabolite candidates. JUMPm identifies all known structures sharing the specified chemical formula. For each candidate structure, the MS/MS fragments are predicted for the theoretical labeled versions using the MetFrag program. Empirical tandem scans are matched and scored against the predicted fragments using the hyper-geometric algorithm, defined as Mscore.

Table S1. List of software tools used in metabolomics data analysis.

Table S1. List of software tools used in metabolomics data analysis

Software tools	Input file format	Data preprocessing	Database searching	Spectral library searching	Labeled data	Unlabeled data	Partial labeled data	Structure	FDR calculation	Publicly available	Graphical user interface (GUI)
XCMS3	mzML	√	√	√		√		√		√	√
SIEVE	Thermo raw	√	√			√					√
Compound Discoverer	Thermo raw	√	√	√	√	√		√			√
Mzmine 2	mzML, mzXML, mzData, NetCDF, Thermo RAW, Waters RAW	√	√			√				√	√
MetAlign	mzData, mzXML, netCDF	√				√				√	√
MAVEN	mzXML	√	√		√	√					√
MS-DIAL	netCDF, mzML	√		√	√			√		√	√
mzMatch	XCMS preprocessed file		√		√	√					√
CAMERA	XCMS preprocessed file				√	√		√			
SIRIUS 4	mgf		√	√		√		√		√	√
MS-DIAL3	netCDF, mzML	√	√	√	√	√	√	√		√	√
JUMPm	mzXML, Thermo raw	√	√	√	√	√	√	√	√	√	
